# CCN4 induces IL-6 production through αvβ5 receptor, PI3K, Akt, and NF-κB singling pathway in human synovial fibroblasts

**DOI:** 10.1186/ar4151

**Published:** 2013-01-23

**Authors:** Chun-Han Hou, Chih-Hsin Tang, Chin-Jung Hsu, Sheng-Mon Hou, Ju-Fang Liu

**Affiliations:** 1 Department of Orthopedic Surgery, National Taiwan University Hospital, 7, Zhongshan South Road, Taipei 100, Taiwan; 2 Department of Pharmacology, School of Medicine, China Medical University, 91 Hsueh-Shih Road, Taichung 402, Taiwan; 3 Graduate Institute of Basic Medical Science, China Medical University, 91 Hsueh-Shih Road, Taichung 402, Taiwan; 4 School of Chinese Medicine, College of Chinese Medicine, China Medical University, 91 Hsueh-Shih Road, Taichung 402, Taiwan; 5 Department of Orthopedic Surgery, China Medical University Hospital, 91 Hsueh-Shih Road, Taichung 402, Taiwan; 6 Department of Orthopedic Surgery, Shin-Kong Wu Ho-Su Memorial Hospital, 95 Wen Chang Road, Taipei 111, Taiwan; 7 Central Laboratory, Shin-Kong Wu Ho-Su Memorial Hospital, 95 Wen Chang Road, Taipei 111, Taiwan

## Abstract

**Introduction:**

Osteoarthritis (OA) is the most common degenerative joint disease that is involved in the degradation of articular cartilage. The exact etiology of OA is not completely understood. CCN4 is related to up-regulation in the cartilage of patients with osteoarthritis. Previous studies have shown that CCN4 might be associated with the pathogenesis of OA, but the exact signaling pathways in CCN4-mediated IL-6 expression in synovial fibroblasts (SF) are largely unknown. Therefore, we explored the intracellular signaling pathway involved in CCN4-induced IL-6 production in human synovial fibroblast cells.

**Methods:**

CCN4-induced IL-6 production was assessed with quantitative real-time qPCR and ELISA. The mechanisms of action of CCN4 in different signaling pathways were studied by using Western blotting. Neutralizing antibodies of integrin were used to block the integrin signaling pathway. Luciferase assays were used to study IL-6 and NF-κB promoter activity. Immunocytochemistry was used to examine the translocation activity of p65.

**Results:**

Osteoarthritis synovial fibroblasts (OASFs) showed significant expression of CCN4 and the expression was higher than in normal SFs. OASF stimulation with CCN4 induced concentration- and time-dependent increases in IL-6 production. Pretreatment of OASFs with αvβ5 but not α5β1 and αvβ3 integrin antibodies reduced CCN4-induced IL-6 production. CCN4-mediated IL-6 production was attenuated by PI3K inhibitor (LY294002 and Wortmannin), Akt inhibitor (Akti), and NF-κB inhibitor (PDTC and TPCK). Stimulation of cells with CCN4 also increased PI3K, Akt, and NF-κB activation.

**Conclusions:**

Our results suggest that CCN4 activates αvβ5 integrin, PI3K, Akt, and NF-κB pathways, leading to up-regulation of IL-6 production. According to our results, CCN4 may be an appropriate target for drug intervention in OA in the future.

## Introduction

Osteoarthritis (OA) is the most common degenerative disease of the synovial joint that involves the degradation of articular cartilage, subchondral bone sclerosis, abnormal bone remodeling, osteophyte formation, and chronic inflammation of the synovial membrane [1,2]. Typical symptoms of OA include joint pain, stiffness, swelling, and muscle weakness. The principal forms of treatment include pain management and replacement surgery. Unfortunately, the exact etiology of OA is not well understood [3]. The synovial membrane is responsible for the inflammatory reaction leading to macrophage-derived proinflammatory cytokines, such as IL-1β, IL-6, IL-8 and TNF-α, that promote inflammation, neovascularization and cartilage degradation via activation of matrix-degrading enzymes, such as matrix metalloproteinases (MMPs) [4-6].

Some evidence suggests that development of OA is often accompanied by inflammation [7,8] and elevated levels of cytokines, such as IL-6 which is a regulator of inflammation in OA. IL-6 has numerous biological activities and is considered as the major player that regulates the innate immune response, haemopoiesis, and inflammation. Numerous studies have demonstrated that IL-6 activates osteoclasts and stimulates the synovium to release MMPs that induce cartilage destruction in OA [9]. A clinical trial showed that IL-6 seems to be the important proinflammatory cytokine involved in the pathophysiology of OA [10,11]. OA patients have a higher concentration of IL-6 in their whole blood compared with that of normal subjects [12]. Similarly, chondrocytes produce low levels of IL-6 under normal conditions. Therefore, these data suggest that IL-6 plays an important role during OA pathogenesis. Several consensus sequences, including those for NF-κB, CREB, NF-IL-6, and AP-1 in the 5'-promoter region of the IL-6 gene, have been identified as regulatory sequences that induce IL-6 in response to various stimuli [13,14]. NF-κB, an important transcription factor that regulates IL-6 expression, is a heterodimer of transcription factor p65 and transcription factor p50. In a resting state, this dimer is associated with IκBs to retain NF-κB in the cytosol. IκB kinase is activated through stimulation by cytokines and bacterial products. Phosphorylation of IκBα at Ser^32 ^and Ser^36 ^and IκBα at Ser^19 ^and Ser^23 ^produces ubiquitination of IκBα/β at lysine residues and degradation by the 26S proteasome [15,16].

WNT-inducible signaling pathway protein-1 (WISP-1, also known as CCN4) belongs to the CCN gene family, which contains cysteine-rich 61 (Cyr61/CCN1), connective tissue growth factor (CTGF/CCN2), nephroblastoma overexpressed (NOV/CCN3), WISP-1/CCN4, WISP-2/CCN5, and WISP-3/CCN6. This gene family encodes secreted proteins that interact with the extracellular matrix and have important roles in migration, adhesion, proliferation, apoptosis, survival, inflammation, and injury repair [17,18]. In a recent study, CCN4 acts in an autocrine manner to accelerate cell growth, induce morphological transformation, increase saturation density, and promote tumorigenesis in normal fibroblasts [19]. In addition, CCN4 is strongly upregulated in the cartilage of patients with OA which can elicit the release of MMPs and aggrecanase from macrophages and chondrocytes which causes cartilage damage [20,21].

Previous studies have shown that CCN4 might be associated with the pathogenesis of OA [20]. However, the role of CCN4 in IL-6 production in osteoarthritis synovial fibroblasts (OASFs) has not been extensively studied. In the present study, we explored the intracellular signaling pathway involved in CCN4-induced IL-6 production in human synovial fibroblast (SF) cells. The results showed that CCN4 activates αvβ5 integrin, PI3K, Akt, and NF-κB pathways, leading to up-regulation of IL-6 expression. According to our results, CCN4 may be an appropriate target for drug intervention in OA in the future.

## Materials and methods

### Materials

Protein A/G beads, anti-mouse and anti-rabbit immunoglobulin G (IgG) conjugated horseradish peroxidase, rabbit polyclonal antibodies specific for PI3K, Akt, IKKα/β, IκB, p65, and β-actin were purchased from Santa Cruz Biotechnology (Santa Cruz, CA, USA). Rabbit polyclonal antibody specific for PI3K phosphorylated at Tyr^458/199^, Akt phosphorylated at ser^473^, IKKα/β phosphorylated at ser^176/180^, IκBα phosphorylated at ser^32/36 ^and p65 phosphorylated at ser^536 ^were purchased from Cell Signaling and Neuroscience (Danvers, MA, USA). Mouse monoclonal antibody specific for αvβ3, αvβ5, and α5β1 integrin were purchased from Chemicon (Temecula, CA, USA). Pyrrolidine-dithiocarbamate (PDTC), L-1-tosylamido-2-phenylenylethyl chloromethyl ketone (TPCK), LY294002, Wortmannin, and pan-Akt inhibitor (1L-6-hydroxymethyl-chiro-inositol-2-((*R*)-2-*O*-methyl-3-*O*-octadecylcarbonate) were purchased from Calbiochem (San Diego, CA, USA). IL-6 enzyme immunoassay kit was purchased from Cayman Chemical (Ann Arbor, MI, USA). The recombinant human CCN4 was purchased from PeproTech (Rocky Hill, NJ, USA). ON-TARGETplus siRNA of αv integrin, β5 integrin, p110 and control were purchased from Dharmacon Research (Lafayette, CO, USA). The NF-κB luciferase plasmid was purchased from Stratagene (La Jolla, CA, USA). The p85 and Akt1 (Akt1 K179A) dominant-negative mutants were gifts from Dr. W.M. Fu (National Taiwan University, Taipei, Taiwan). The human IL-6 promoter construct pIL6-luc651 (-651/+1), AP-1 site mutation (pIL6-luc651ΔAP1), NF-κB site mutation (pIL6-luc651ΔNF-κB), and C/EBP-β site mutation (pIL6-luc651ΔC/EBP-β) [22] were gifts from Dr. Oliver Eickelberg (Ludwig Maximilians University Munich, Munich, Germany). The pSV-β-galactosidase vector and the luciferase assay kit were purchased from Promega (Madison, MA, USA). All other chemicals were purchased from Sigma-Aldrich (St. Louis, MO, USA).

### Cell cultures

Written informed consent was obtained from all patients and the study was approved by the Institutional Review Board of Shin Kong Wu Ho-Su Memorial Hospital. Human SFs were isolated by collagenase treatment of synovial tissue samples obtained from 32 patients with OA during knee-replacement surgeries and 18 samples of nonarthritic synovial tissues obtained at arthroscopy after trauma/joint derangement. Fresh synovial tissues were minced and digested in a solution of collagenase and DNase. Isolated fibroblasts were filtered through 70-µm nylon filters. The cells were grown on plastic cell culture dishes in 95% air-5% CO2 with (D)MEM (Life Technologies, Grand Island, NY, USA) that was supplemented with 20 mM HEPES and 10% heat-inactivated fetal bovine serum (FBS), 2 mM glutamine, 100 U/ml penicillin, and 100 µg/ml streptomycin (pH adjusted to 7.6). Fibroblasts from passages four to nine were used for the experiments [23,24]. The cells from different patients were treated with CCN4 separately. All studies were carried out on cells from at least four patients. Results are from four independent experiments [2].

### Measurement of IL-6 production

Human SFs were cultured in 24-well culture plates. After reaching confluence, cells were treated with CCN4 and then incubated in a humidified incubator at 37°C for 24 hours. For examination of the downstream signaling pathways involved in CCN4 treatment, cells were pretreated with various inhibitors for 30 minutes (these inhibitors did not affect cell viability; Additional file 1, Figure S1) before CCN4 (30 ng/ml) administration. After incubation, the medium was removed and stored at -80°C until assay. IL-6 in the medium was assayed using the IL-6 enzyme immunoassay kits, according to the procedure described by the manufacturer.

### Quantitative real-time PCR

Total RNA was extracted from SFs using a TRIzol kit (MDBio Inc., Taipei, Taiwan). The reverse transcription reaction was performed using 2 µg of total RNA that was reverse transcribed into cDNA using oligo (dT) primer [25,26]. The quantitative real-time PCR (qPCR) analysis was carried out using Taqman® one-step PCR Master Mix (Applied Biosystems, Foster City, CA). cDNA templates (2 µl) were added per 25-µl reaction with sequence-specific primers and Taqman® probes. Sequences for all target gene primers and probes were purchased commercially (β-actin was used as internal control) (Applied Biosystems). The qPCR assays were carried out in triplicate on a StepOnePlus sequence detection system. The cycling conditions were 10-minute polymerase activation at 95°C, followed by 40 cycles at 95°C for 15 seconds and 60°C for 60 seconds. The threshold was set above the non-template control background and within the linear phase of the target gene amplification to calculate the cycle number at which the transcript was detected (denoted CT) [27].

### Western blot analysis

Cellular lysates were prepared as described previously [28,29]. Proteins were resolved on SDS-PAGE and transferred to immobilon polyvinyldifluoride (PVDF) membranes. The blots were blocked with 4% BSA for 1 hour at room temperature and then probed with rabbit anti-human antibodies against PI3K, Akt, IKKα/β and IκB, p65, (1:1000) for 1 hour at room temperature. After three washes, the blots were subsequently incubated with donkey anti-rabbit peroxidase-conjugated secondary antibody (1:3000) for 1 hour at room temperature. The blots were visualized by enhanced chemiluminescence with Kodak X-OMAT LS film (Eastman Kodak, Rochester, NY, USA). Quantitative data were obtained using a computing densitometer and ImageQuant software (Molecular Dynamics, Sunnyvale, CA, USA).

### Transfection and reporter gene assay

Human SFs were co-transfected with 0.8 µg luciferase plasmid and 0.4 µg β-galactosidase expression vector. Fibroblasts were grown to 80% confluence in 12 well plates and were transfected the following day with Lipofectamine 2000 (LF2000; Invitrogen, Carlsbad, CA, USA). DNA and LF2000 were premixed for 20 minutes and then applied to the cells (the transfection efficiency is more than 90%, confirmed by transfection with Green fluorescent protein (GFP) -expressing plasmid; Additional file 1, Figure S2). After 24 hours transfection, cells were incubated with the indicated agents. After a further 24-hour incubation, the media were removed and the cells were washed once with cold PBS. To prepare lysates, 100 µl reporter lysis buffer (Promega) was added to each well, and the cells were scraped from the dishes. The supernatant was collected after centrifugation at 13,000 rpm for two minutes. Aliquots of cell lysates (20 µl) containing equal amounts of protein (20 µg to 30 µg) were placed into wells of an opaque black 96-well microplate. An equal volume of luciferase substrate was added to all samples and luminescence was measured in a microplate luminometer. The value of luciferase activity was normalized to transfection efficiency monitored by the co-transfected β-galactosidase expression vector [30].

### Immunofluorescence staining

Cells were cultured in 12 mm coverslips. After treatment with CCN4, cells were fixed with 4% paraformaldehyde at room temperature. Thirty minutes later, 4% nonfat milk in PBS containing 0.5% Triton X-100 was added to the cells. The cells were then incubated with rabbit anti-p65 (1:100) and fluorescein isothiocyanate (FITC)-conjugated goat anti-rabbit secondary antibody (1:500; Leinco Technology Inc., St Louis, MO, USA) for 1 hour, respectively. The FITC was detected using a Zeiss fluorescence microscope [31].

### Statistics

The values reported are means ± standard error of the mean (SEM). Statistical analysis between two samples was performed using Student's *t-*test. Statistical comparisons of more than two groups were performed using one-way analysis of variance (ANOVA) with Bonferroni's *post-hoc *test. In all cases, *P *<0.05 was considered significant.

## Results

### CCN4 induces IL-6 production in human synovial fibroblasts

**Figure 1:**
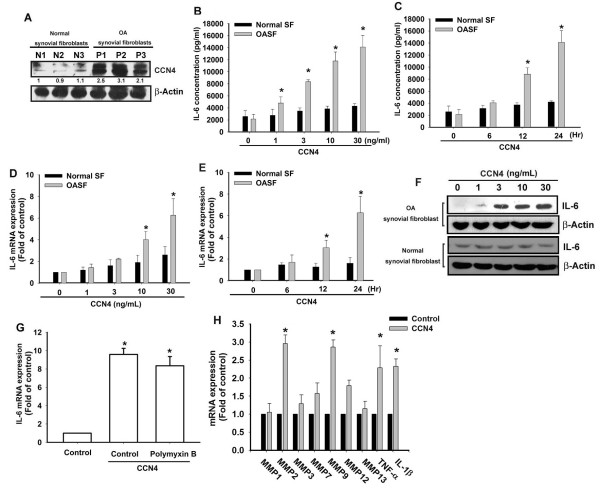
**Concentration- and time-dependent increases in IL-6 production by CCN4**. (**A**) Human synovial fibroblasts (SFs) were obtained from normal (*n *= 3) or osteoarthritis patients (*n *= 3) and examined with western blotting for the expression of CCN4. (**B, C**) OASFs and normal SFs were incubated with various concentrations of CCN4 for 24 hours or with CCN4 (30 ng/ml) for 6, 12, or 24 hours. Media were collected to measure IL-6 production (*n *= 4). (**D, E**) OASFs and normal SFs were incubated with various concentrations of CCN4 for 24 hours or with CCN4 (30 ng/ml) for 6, 12, or 24 hours. The mRNA expression of IL-6 was examined by qPCR (*n *= 4). (**F**) OASFs and normal SFs were incubated with various concentrations of CCN4 for 24 hours, and the protein expression of IL-6 was examined by Western blot (*n *= 3). (**G**) OASFs were pretreated with polymyxin B (poly B, 1 μM) for 30 minutes followed by stimulation with CCN4 (30 ng/ml), and IL-6 expression was examined by qPCR (*n *= 3). (**H**) OASFs were incubated with of CCN4 (30 ng/ml), and the mRNA expression was examined by qPCR (*n *= 3). Results are expressed as the mean ± SEM. *, *P *<0.05 compared with control; #, *P *<0.05 compared with CCN4-treated group. OASFs, osteoarthritis synovial fibroblasts; SEM, standard error of the mean.

It has been reported that CCN4 plays an important role during OA pathogenesis. [21]. First, we examined human SF tissues for the expression of CCN4 by using Western blotting. The expression of CCN4 in patients with OA was significantly higher than in controls (Figure 1A). Next, we directly applied CCN4 in OASFs and examined the expression of IL-6 (an important inflammatory response gene). Treatment of OASFs with CCN4 (1 to 30 ng/ml) for 24 hours induced IL-6 production in a concentration-dependent manner (Figure 1B), and this induction occurred in a time-dependent manner (Figure 1C). In addition, stimulation of cells with CCN4 also increased mRNA expression of IL-6 in a concentration- and time- dependent manner (Figure 1D and 1E). On the other hand, treatment of OASF cells with CCN4 (1 to 30 ng/ml) for 24 hours also induced IL-6 protein expression (Figure 1F). To confirm this stimulation specific mediation by CCN4 without endotoxin contamination, polymyxin B, a lipopolysaccharide inhibitor, was used. We found that polymyxin B (1 μM) did not affect CCN4-induced IL-6 expression (Figure 1G). Therefore, CCN4 used in this study is endotoxin free. In contrast, CCN4 did not affect IL-6 expression in normal SFs (Figure 1B to 1F). Therefore, OASFs are more sensitive to CCN4 than normal SFs. We also investigated whether CCN4 induced other inflammatory cytokines (TNF-α and IL-1β) and MMPs expression in human SFs. Stimulation of cells with CCN4 increased MMP-2 and -9 but not -1, -3, -7,-12, and -13 expression (Figure 1H). In addition, CCN4 also enhanced the expression of TNF-α and IL-1β in human SFs (Figure 1H).

### Involvement of αvβ5 integrin in CCN4 induced IL-6 production

**Figure 2:**
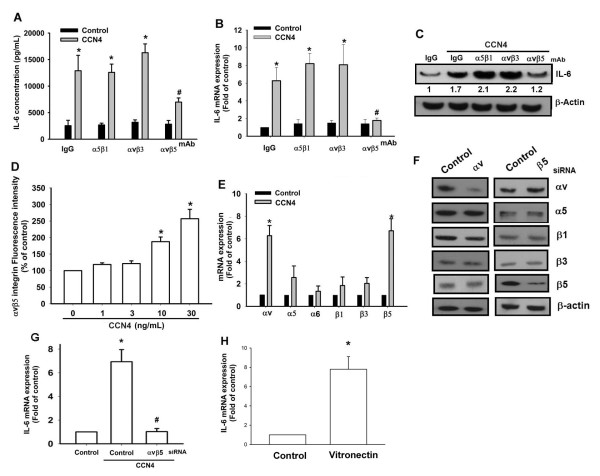
**αvβ5 integrin is involved in CCN4-mediated IL-6 production in synovial fibroblast**.**s **(**A-C**) OASFs were pretreated for 30 minutes with α5β1, αvβ3, or αvβ5 integrin antibody (3 µg/ml) followed by stimulation with CCN4 for 24 hours, and IL-6 expression was examined by qPCR, ELISA, and Western blot. *, *P *<0.05 compared with IgG control. (**D**) OASFs were incubated with various concentrations of CCN4 for 24 hours, and the cell surface αvβ5 integrin expression was examined by flow cytometry. (**E**) OASFs were incubated with CCN4 (30 ng/ml) for 24 hours, and the mRNA expression of αv and β5 integrins was examined by qPCR. (**F**) OASFs were transfected for 24 hours with αv and β5 siRNA, and the integrins expression was examined by Western blot. (**G**) OASFs were cotransfected for 24 hours with αv and β5 siRNA followed by stimulation with CCN4 for 24 hours, and IL-6 expression was examined by qPCR. (**H**) OASFs were incubated with vitronectin (10 ng/ml) for 24 hours, and IL-6 expression was examined by qPCR. Results are expressed as the mean ± SEM (*n *= 3). *, *P *<0.05 compared with control; #, *P *<0.05 compared with the CCN4-treated group. OASFs, osteoarthritis synovial fibroblasts; IgG, immunoglobulin G; SEM, standard error of the mean.

Previous study has shown that the CCN family affects cell functions by binding to cell surface integrin receptors [32]. Therefore, we hypothesized that the integrin signaling pathway might be involved in CCN4-induced IL-6 production. Pretreatment of OASFs with αvβ5 integrin monoclonal antibody (mAb; 5 µg/ml) for 30 minutes markedly inhibited CCN4-induced IL-6 production (Figure 2A), whereas α5β1 and αvβ3 integrin receptor-specific mAb or control IgG did not have the same effect. In addition, pretreatment of OASF cells with αvβ5 integrin mAb reduced CCN4-induced IL-6 expression (Figure 2B, C). To further confirm CCN4 induced IL-6 production through αvβ5 integrin, we then examined αvβ5 integrin expression after CCN4 treatment. Stimulation of OASFs increased cell surface and mRNA expression of αv and β5 integrin (Figure 2D, E), suggesting that the amplification loop strengthens the CCN4-integrin-signaling pathway. To rule out that the increasing IL-6 production is induced by upregulation of αvβ5 and not CCN4, the ON-TARGETplus αv and β5 siRNA were used (the design of ON-TARGETplus siRNA can prevent off-target effects of siRNA). Transfection αv and β5 siRNA reduced αv and β5 integrin expression, respectively (Figure 2F). In addition, cotransfection αv and β5 siRNA completely reduced CCN4-mediated IL-6 expression (Figure 2G). Therefore, the induced CCN4 expression is due to CCN4 but not upregulation of αvβ5. In addition, treatment of OASFs with vitronectin (αvβ5 integrin ligand) also increased IL-6 expression (Figure 2H). Therefore, activation of αvβ5 integrin plays a key role in IL-6 expression in human SFs.

### PI3K/Akt signaling pathway is involved in CCN4-mediated IL-6 production

**Figure 3:**
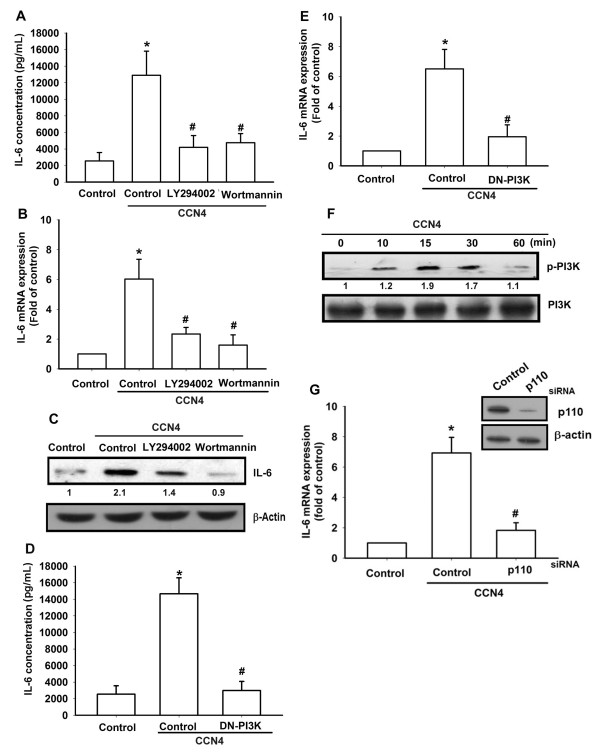
**PI3K is involved in CCN4-mediated IL-6 production in synovial fibroblasts**. (**A-C**) OASFs were pretreated for 30 minutes with LY294002 (10 µM) and Wortmannin (5µM) followed by stimulation with CCN4 for 24 hours. Media, total RNA, and protein were collected, and the expression of IL-6 was analyzed by qPCR, ELISA, and Western blot. (**D, E**) OASFs were transfected for 24 hours with PI3K mutant followed by stimulation with CCN4 for 24 hours. Media and total RNA were collected, and the expression of IL-6 was analyzed by qPCR and ELISA. (**F**) Cells were incubated with CCN4 for the indicated time intervals, and PI3K phosphorylation was examined by Western blot. Results are expressed as the mean ± SEM (*n *= 3). *, *P *<0.05 compared with control; #, *P *<0.05 compared with the CCN4-treated group. OASFs, osteoarthritis synovial fibroblasts; SEM, standard error of the mean.

**Figure 4:**
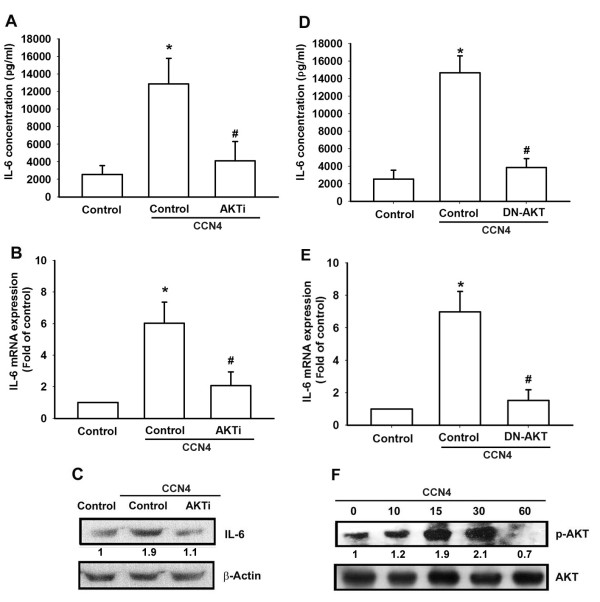
**Akt is involved in the potentiation of IL-6 production by CCN4**. (**A-C**) OASFs were pretreated for 30 minutes with Akti (10 µM) followed by stimulation with CCN4 for 24 hours. Media, total RNA and protein were collected, and the expression of IL-6 was analyzed by qPCR, ELISA, and Western blot. (**D, E**) OASFs were transfected for 24 hours with Akt mutant followed by stimulation with CCN4 for 24 hours. Media and total RNA were collected, and the expression of IL-6 was analyzed by qPCR and ELISA. (**F**) Cells were incubated with CCN4 for the indicated time intervals, and Akt phosphorylation was examined by Western blot. Results are expressed as the mean ± SEM (*n *= 4). *, *P *<0.05 compared with control; #, *P *<0.05 compared with the CCN4-treated group. OASFs, osteoarthritis synovial fibroblasts; SEM, standard error of the mean.

The PI3K/Akt signaling pathway can be activated by a variety of factors including insulin and different growth factors [33,34]. It has been reported that PI3K and Akt activation regulates the IL-6 expression in OASFs [35]. We then investigated the role of PI3K in mediating CCN4-induced IL-6 expression using the specific PI3K inhibitors LY294002 and Wortmannin. As shown in Figure 3A-C, CCN4-induced IL-6 expression was markedly attenuated by pretreatment with LY294002 and Wortmannin for 30 minutes in OASFs. The viability of the human osteosarcoma cell line MG63 was not affected by LY294002 and Wortmannin as shown by the 3-(4,5-dmethylthiazol-2-yl)-2,5-diphenyltetrazolium bromide (MTT) assay (see Additional file 1, Figure S1). In addition, transfection of OASFs with PI3K mutant also reduced CCN4-increased IL-6 production (Figure 3D, E). We then directly measured p85 phosphorylation in response to CCN4. Stimulation of cells led to a significant increase of phosphorylation of p85 (Figure 3F). p85 is one of the regulatory subunits of PI3K. In contrast, p110 is a catalytic subunit of PI3K. Transfection cells with p110 siRNA reduced p110 expression and also reduced CCN4-induced IL-6 expression (Figure 3G). Therefore, blocked each regulatory or catalytic subunit of PI3K can abolished CCN4-meidated IL-6 expression. Furthermore, to examine the crucial role of Akt signaling in CCN4-induced IL-6 production, we pretreated cells with Akt inhibitor and antagonized CCN4-increased IL-6 production (Figure 4A-C). The viability of the human osteosarcoma cell line MG63 was not affected by AKTi as shown by the MTT assay [see Additional file 1, Figure S1]. In addition, the Akt mutant also inhibited CCN4-increased IL-6 production (Figure 4D, E). Akt phosphorylation at Ser^473 ^by a PI3K-dependent signaling pathway causes enzymatic activation [36]. We directly measured Akt phosphorylation in response to CCN4. Treatment of cells with CCN4 resulted in time-dependent phosphorylation of Akt Ser^473 ^(Figure 4F). Based on these results, it appears that CCN4 acts through the PI3K and Akt-dependent signaling pathway to enhance IL-6 production in OASFs.

### Involvement of NF-κB in CCN4 induced IL-6 production

**Figure 5:**
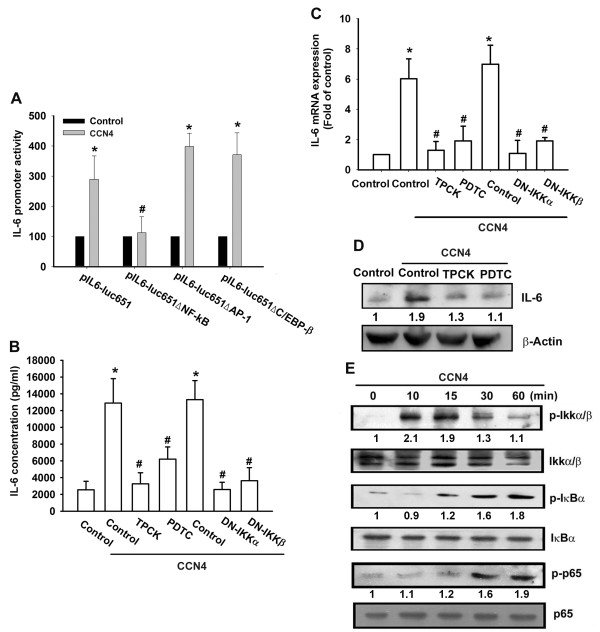
**NF-κB is involved in the potentiation of IL-6 production by CCN4**. **(A**) OASFs were transfected with IL-6 luciferase plasmids before incubation with CCN4 for 24 hours. Luciferase activity was then assayed. (**B-D**) OASFs were pretreated for 30 minutes with PDTC (10 µM) and TPCK (10 µM) or transfected for 24 hours with IKKα and IKKβ mutant followed by stimulation with CCN4 for 24 hours. Media, total RNA, and total protein were collected to measure IL-6 by ELISA, qPCR, and Western blot. (**E**) OASFs were incubated with CCN4 for the indicated time intervals, and p-IKKα/β, p-IκBα, and p-p65 expression was determined by Western blot analysis. Results are expressed as the mean ± SEM (*n *= 3). *, *P *<0.05 compared with control; #, *P *<0.05 compared with CCN4-treated group. OASFs, osteoarthritis synovial fibroblasts; PDTC, pyrrolidine-dithiocarbamate; SEM, standard error of the mean, TPCK, L-1-tosylamido-2-phenylenylethyl chloromethyl ketone.

The promoter region of human IL-6 contains three known cis-regulatory elements including AP-1, C/EBP-β, and NF-κB binding sites [13,14]. Three different IL-6 promoter constructs containing mutations at NF-κB, AP-1, or C/EBP-β sites, respectively, were generated by site-directed mutagenesis. We found that CCN4-stimulated IL-6 luciferase activity was abolished by NF-κB-binding site mutation, but not by AP-1 or C/EBP-β site mutations (Figure 5A). To examine whether NF-κB activation is involved in the signal transduction pathway leading to IL-6 production caused by CCN4, the NF-κB inhibitor PDTC was used. Pretreatment of cells with PDTC (5 µM) reduced CCN4-increased IL-6 expression (Figure 5B-D). Furthermore, the IκB protease inhibitor TPCK (5 µM) also antagonized the potentiating action of IL-6 (Figure 5B-D). Therefore, the NF-κB binding site is more important than the AP-1 and C/EBP-β sites in CCN4-induced IL-6 production.

**Figure 6:**
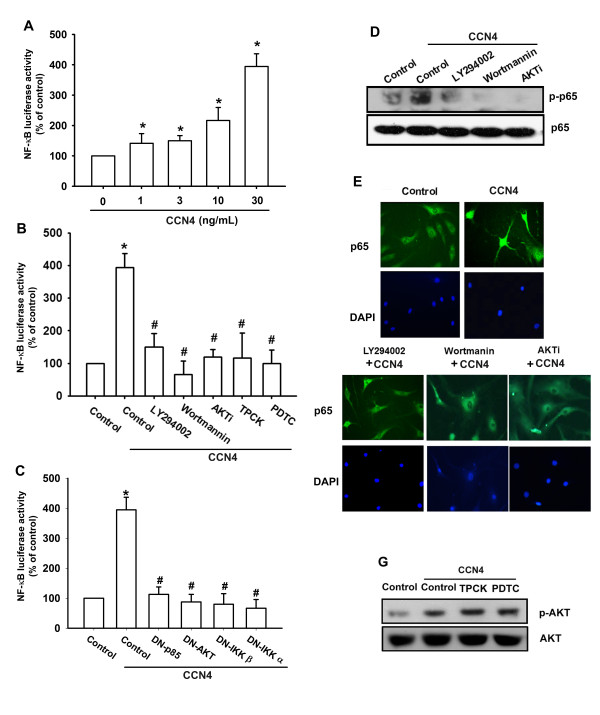
**CCN4 induced NF-κB activation through αvβ5 integrin/PI3K/Akt pathway**. (**A-C**) OASFs were incubated with various concentrations of CCN4 or pretreated with LY294002, Wortmannin or Akti, PDTC or TPCK for 30 minutes or transfected with PI3K, Akt, IKKα, and IKKβ mutant before exposure to CCN4. NF-κB luciferase activity was measured, and the results were normalized to the β-galactosidase activity. (**D**) OASFs were pretreated with LY294002, Wortmannin or Akti for 30 minutes followed by stimulation with CCN4 for 60 minutes, and p-p65 expression was examined by Western blot analysis. (**E**) OASFs were pretreated with LY294002, Wortmannin or Akti for 30 minutes then stimulated with CCN4 for 120 minutes, and p65 immunofluorescence staining was examined. (**F**) OASFs were pretreated with PDTC and TPCK for 30 minutes followed by stimulation with CCN4 for 30 minutes, and p-Akt expression was examined by Western blot analysis. Results are expressed as the mean ± SEM (*n *= 4). *, *P *<0.05 compared with control; #, *P *<0.05 compared with the CCN4-treated group. OASFs, osteoarthritis synovial fibroblasts; PDTC, pyrrolidine-dithiocarbamate; SEM, standard error of the mean, TPCK, L-1-tosylamido-2-phenylenylethyl chloromethyl ketone.

We further examined the upstream molecules involved in CCN4-induced NF-κB activation. Stimulation of cells with CCN4 induced IKKα/β phosphorylation in a time-dependent manner (Figure 5E). Transfection of cells with IKKα or IKKβ mutant reduced CCN4-increased IL-6 production (Figure 5B, C). In addition, treatment of cells with CCN4 also caused IκBα phosphorylation in a time-dependent manner (Figure 5E). Previous studies showed that p65 Ser^536 ^phosphorylation increases NF-κB transactivation [37]. Therefore, the antibody specific against phosphorylated p65 Ser^536 ^was employed to examine p65 phosphorylation. Treatment of cells with CCN4 for various time intervals resulted in p65 Ser^536 ^phosphorylation (Figure 5E). To further confirm that the NF-κB element is involved in the action of CCN4 induced IL-6 expression, we performed transient transfection using the NF-κB promoter-luciferase constructs. OASFs incubated with CCN4 led to an increase in NF-κB promoter activity in a dose dependent manner (Figure 6A). The increase of NF-κB activity by CCN4 was antagonized by LY294002, Wortmannin, Akti, TPCK, PDTC, or p85, Akt, IKKα, and IKKβ mutant (Figure 6B, C). Furthermore, LY294002, Wortmannin and Akti also reduced CCN4-mediated p65 phosphorylation and translocation into the nucleus (Figure 6D, E). To examine whether inhibition of NF-κB pathway affects Akt signing in CCN4 treated cells, OASFs were incubated with PDTC and TPCK, and Akt phosphorylation was examined. We found that PDTC and TPCK did not affect CCN4-induced Akt activation (Figure 6F). Taken together, these data suggest that activation of the αvβ5 integrin, PI3K, Akt, and NF-κB pathway is required for the CCN4-induced increase of IL-6 in human SFs.

## Discussion

In addition to related changes in the underlying bone and joint, OA is mentioned as a heterogeneous group of conditions that are associated with defective integrity of articular cartilage [28]. Chronic inflammation of the synovial membrane contributes to the development of disease and cartilage degradation through a complex cytokine network. All the factors responsible for initiating the degradation and loss of the articular tissues are not yet completely understood. CCN proteins are considered to play a pivotal role in inflammation [38,39]. It has been recently reported that CCN4 is involved in the pathogenesis of arthritis, because CCN4 was able to elicit the release of MMPs and aggrecanase from macrophages and chondrocytes to cause cartilage damage [20,21,40]. Although the involvement of CCN4 in inflammatory responses has been reported, the molecular mechanisms underlying CCN4 in IL-6 expression in OA are still unclear. In this study, we further demonstrated that IL-6 is a target protein for the CCN4 signaling pathway that regulates the cell inflammatory response. Our results also indicated that CCN4 induced IL-6 synthesis occurs at 12 hours (Figure 1). However, stimulation of the cells with CCN4 for 12 hours only increased IL-6 mRNA expression but not TNF-α and IL-1β [see Additional file 1, Figure S3]. Therefore, CCN4-induced IL-6 expression is the direct result of CCN4 but the other cytokines did not respond to CCN4. We also showed that potentiation of IL-6 by CCN4 requires activation of the αvβ5 integrin receptor, PI3K, Akt, and NF-κB signaling pathways. These findings suggest that CCN4 in OA acts as an inducer of inflammatory cytokines, such as IL-6 and enhances the inflammatory response.

CCN4 is known to activate integrin, including α5β1, αvβ3, and αvβ5 [41,42]. However, we demonstrated that αvβ5 integrin but not α5β1 and αvβ3 integrin receptor was required for CCN4-induced IL-6 production. Treatment of cells with αvβ5, but not α5β1 and αvβ3 integrin mAb, inhibited CCN4-induced IL-6 production. In addition, stimulation of OASFs with CCN4 increased the cell surface and mRNA expression of αv and β5 integrin. These data suggest that αvβ5 integrin is involved in CCN4-induced IL-6 production in human SFs.

It has been reported that a variety of growth factors stimulate the expression of IL-6 genes via signal-transduction pathways that converge to activate the NF-κB complex of transcription factors and that PI3K/Akt were involved in the activation of NF-κB transcription factors. However, we demonstrated that CCN4 enhanced PI3K and Akt phosphorylation in human OASFs. Previous studies have revealed that CCN4 is a potent activator of PI3K/Akt, the critical players in cell survival and growth [43,44]. The CCN4-directed IL-6 production was effectively inhibited by PI3K or Akt inhibitor. This was further confirmed by the results that showed that the dominant negative mutant of PI3K and Akt inhibited the enhancement of IL-6 production by CCN4. Our data indicate that PI3K/Akt may play an important role in the expression of IL-6 in human OASFs. The MAPK pathway is a common targeting molecule in integrin-dependent signaling [45]. However, pretreatment of cells with JNK inhibitor (SP600125) and p38 inhibitor (SB203580) but not ERK inhibitor (PD98059) reduced CCN4-mediated IL-6 expression [see Additional file 1, Figure S4]. Therefore, JNK and p38 but not ERK are also involved in CCN4-induced IL-6 expression in human SFs.

There are several binding sites for a number of transcription factors including NF-κB, CREB, NF-IL-6, and the AP-1 box in the 5' region of the IL-6 gene [13,14]. Recent studies on the IL-6 promoter have demonstrated that IL-6 is induced by several transcription factors in a highly stimulus-specific or cell-specific manner. The results of this study show that NF-κB activation contributes to CCN4-induced IL-6 production in SFs, and deletion of the NF-κB site reduced CCN4-mediated IL-6 promoter activity. Pretreatment of cells with NF-κB inhibitors (PDTC and TPCK) also reduced CCN4-increased IL-6 production. Therefore, the NF-κB-binding site is important in CCN4-induced IL-6 production. It is established that the NF-κB sequence binds to members of the p65 and p50 families of transcription factors, and the results of this study show CCN4-induced p65 phosphorylation and nuclear accumulation. Furthermore, using transient transfection with NF-κB-luciferase as an indicator of NF-κB activity showed that CCN4 increased NF-κB activation. In addition, LY294002, Wortmannin, and Akti or PI3K and Akt mutant reduced CCN4-increased NF-κB promoter activity. These results indicate that CCN4 increased NF-κB activation through the αvβ5 integrin/PI3K/Akt signaling pathway in human OASFs.

## Conclusions

In conclusion, we explored the signaling pathway involved in CCN4-induced IL-6 production in human SFs. We found that CCN4 increases IL-6 production by binding to the αvβ5 integrin and activating PI3K/Akt signaling which enhances NF-κB transcription activity and results in the transactivation of IL-6 production. Furthermore, the discovery of a CCN4/αvβ5 integrin-mediated signaling pathway helps us understand the mechanism of OA pathogenesis and may lead us to develop effective therapy in the future.

## Abbreviations

BSA: bovine serum albumin; CTGF: connective tissue growth factor; Cyr61: cysteine-rich 61; (D)MEM: (Dulbecco's) modified Eagle's medium; ELISA: enzyme-linked immunosorbent assay; FBS: fetal bovine serum; FITC: fluorescein isothiocyanate; IgG: immunoglobulin G; IL: interleukin; mAb: monoclonal antibody; MMP: matrix metalloproteinase; MTT: 3-(4,5-dmethylthiazol-2-yl)-2,5-diphenyltetrazolium bromide; NOV: nephroblastoma overexpressed; OA: osteoarthritis; OASFs: osteoarthritis synovial fibroblasts; PBS: phosphate buffered saline; PDTC: pyrrolidine dithiocarbamate; qPCR: quantitative real-time polymerase chain reaction; SEM: standard error of the mean; TNF: tumor necrosis factor; TPCK: L-1-tosylamido-2-phenylenylethyl chloromethyl ketone; WISP-1: WNT-inducible signaling pathway protein-1.

## Competing interests

The authors declare that they have no competing interests.

## Authors' contributions

JFL and SMH conceived and designed the experiments. CHH, CHT, CJH and JFL performed the experiments. CHH, CHT, SMH and JFL analyzed the data. CHH, CHT, SMH and JFL contributed reagents/materials/analysis tools. JFL and SMH wrote the paper. All authors read and approved the final manuscript.

## Supplementary Material

Additional file 1 **The cell viability of PI3K, Akt, and NF-κB inhibitors in human synovial fibroblasts**. OASFs were treated with Wortmannin, Ly294002, Akt inhibitor, PDTC, or TPCK for 24 hours. The cell viability was examined by MTT assay. MTT, 3-(4,5-dmethylthiazol-2-yl)-2,5-diphenyltetrazolium bromide; OASFs, osteoarthritis synovial fibroblasts; PDTC, pyrrolidine-dithiocarbamate; TPCK, L-1-tosylamido-2-phenylenylethyl chloromethyl ketone. Click here for file
