# Supplementary material for: CCN4 induces IL-6 production through αvβ5 receptor, PI3K, Akt, and NF-κB singling pathway in human synovial fibroblasts
Source: Arthritis Res Ther. 2013 Jan 23;15(1):R19. doi: 10.1186/ar4151 (PMC3672729; doi:10.1186/ar4151)
Supplement: Additional file 1 — The cell viability of PI3K, Akt, and NF-κB inhibitors in human synovial fibroblasts. OASFs were treated with Wortmannin, Ly294002, Akt inhibitor, PDTC, or TPCK for 24 hours. The cell viability was examined by MTT assay. MTT, 3-(4,5-dmethylthiazol-2-yl)-2,5-diphenyltetrazolium bromide; OASFs, osteoarthritis synovial fibroblasts; PDTC, pyrrolidine-dithiocarbamate; TPCK, L-1-tosylamido-2-phenylenylethyl chloromethyl ketone. [file ar4151-S1.DOC]

**Supplementary** **Data**


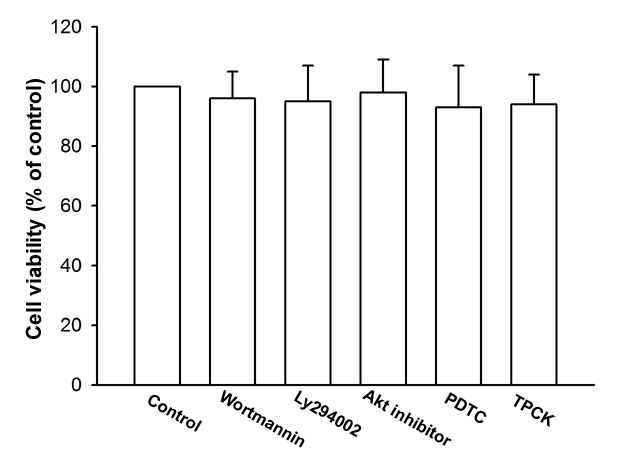


**Fig. S1. The cell viability of PI3K, Akt, and NF-κB inhibitors in human synovial fibroblasts.** OASFs were treated with Wortmannin, Ly294002, Akt inhibitor, PDTC, or TPCK for 24 h. The cell viability was examined by MTT assay. Results are expressed as the mean ± S.E.

**
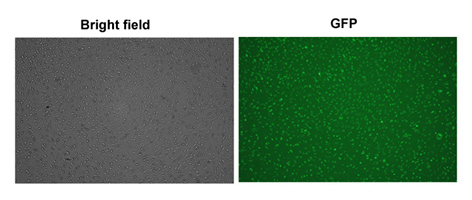
**

**Fig. S2. The transfection efficiency in human synovial fibroblasts.** OASFs were transfected with GFP-expressing plasmid for 24 h, the GFP fluorescence staining was examined. The results indicated that the transfection efficiency is more than 90%.


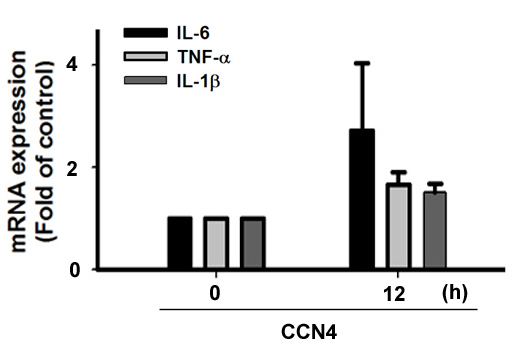


**Fig. S3. The mRNA expression of IL-6, TNF-α and IL-1β after CCN4 treatment.** OASFs were treated with CCN4 for 24 h, the mRNA expression was examined by qPCR. Results are expressed as the mean ± S.E.


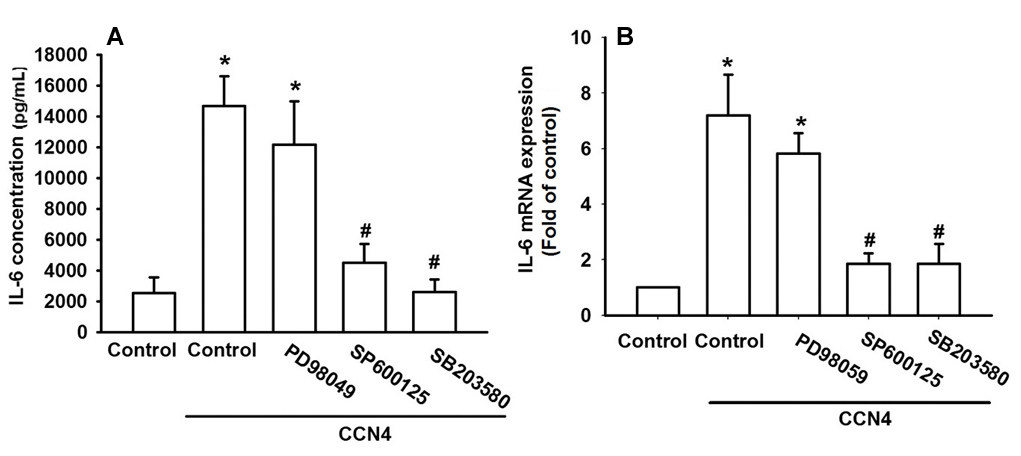


**Fig. S4. JNK and p38 but not ERK are involved in CCN4-induced IL-6 expression.** OASFs were pretreated for 30 min with JNK inhibitor (SP600125), p38 inhibitor (SB203580) and ERK inhibitor (PD98059) for 30 min followed by stimulation with CCN4 for 24 h. Media and total RNA were collected, and the expressions of IL-6 were analyzed by ELISA and qPCR. Results are expressed as the mean ± S.E. *, p < 0.05 compared with control; #, p < 0.05 compared with CCN4-treated group
